# 6-Methoxy Podophyllotoxin Induces Apoptosis via Inhibition of *TUBB3* and *TOPIIA* Gene Expressions in 5637 and K562 Cancer Cell Lines

**DOI:** 10.22074/cellj.2015.10

**Published:** 2015-10-07

**Authors:** Iman Sadeghi, Mehrdad Behmanesh, Najmeh Ahmadian Chashmi, Mohsen Sharifi, Bahram Mohammad Soltani

**Affiliations:** 1 Department of Genetics, Faculty of Biological Sciences, Tarbiat Modares University, Tehran, Iran; 2 Department of Plant Biology, Faculty of Biological Sciences, Tarbiat Modares University, Tehran, Iran

**Keywords:** Podophyllotoxin, Cancer, Apoptosis, Gene Expression, Real-Time PCR

## Abstract

**Objective:**

Podophyllotoxin (PTOX), a natural compound in numerous plants, contains remarkable biological properties that include anti-tumor, anti-viral such as anti-human im- munodeficiency virus (HIV) activities. In order to avoid its adverse effects, various com- pounds have been derived from PTOX. 6-methoxy PTOX (MPTOX) is one of the natural PTOX derivatives with an extra methoxy group. MPTOX is mostly isolated from the Linum species. This study has sought to determine the biological effects of MPTOX on cancer cell lines, 5637 and K562.

**Materials and Methods:**

In this experimental study, we treated the 5637 and K562 cancer cell lines with MPTOX in a doseand time-dependent manner. Apoptosis was examined by flow cytometry and viability rate was analyzed by the MTT assay. Expressions of the tubulin (*TUBB3*) and topoisomerase II (*TOPIIA*) genes were determined by real-time poly- merase chain reaction (PCR).

**Results:**

Treatment with MPTOX led to significant induction of apoptosis in cancer cells compared to control cells. Gene expression analysis showed reduced levels of *TUBB3* and *TOPIIA* mRNA following MPTOX treatment.

**Conclusion:**

MPTOX inhibited *TUBB3* and *TOPIIA* gene expression and subsequently induced cell death through apoptosis. These results suggested that MPTOX could be considered a potential anti-tumor agent.

## Introduction

Chemotherapy, one of the most common treatment for cancers, is comprised of different natural and synthetic compounds. Among these, plant compounds have been used for an extended period of time. Lignan-containing plants are of interest in the Eastern world throughout a number of centuries; they have a wide variety of biological activities which include anti-cancer properties of the aryl tetralinlignans (1, 2).

Podophyllotoxin (PTOX) is a member of the lignan family extracted from the genera Podophyllum (3). Several PTOX derivatives have been isolated from these plants. According to recent research, the Linum species are additional sources for these cytotoxic lignans (4). PTOX has been initially used as treatment for genital warts (5) as it can inhibit the growth of epithelial cells infected by the human papilloma virus (HPV) in the epidermis (6). Also, it is the pharmacological precursor of etoposide and teniposide (7, 8), both of which are important anti-cancer compounds.

Etoposide and teniposide are used to treat various types of cancers such as small cell lung cancer (9), testicular carcinoma (10), and lymphomas (11). The mechanism of action for PTOX-like compounds is the same as colchicine. Both inhibittubulin polymerization andmitotic spindle formation, resulting in thearrest of the cell division process during metaphase (12, 13).

PTOX-like compounds inhibit tubulin polymerization. However, due to an additional glucoside branches, they also inhibit microtubuleassembly (14). It is known that the etoposide effects on DNA topoisomerase II, which are re - quired for correction of topology and conformation of DNA structure. Topoisomerase II (topoisomerase (DNA) II alpha (TopIIA)) is a 170 KDa enzyme that regulates the over-winding or under-winding of DNA, chromosome condensation, and chromatid separation, hence it is essential for DNA replication. Chemotherapy drugs such as topoisomerase inhibitors work by interfering with topoisomerases in cancer cells. This interference induces breaks in the DNA, which ultimately lead to programmed cell death (15, 16).

**Fig.1:**
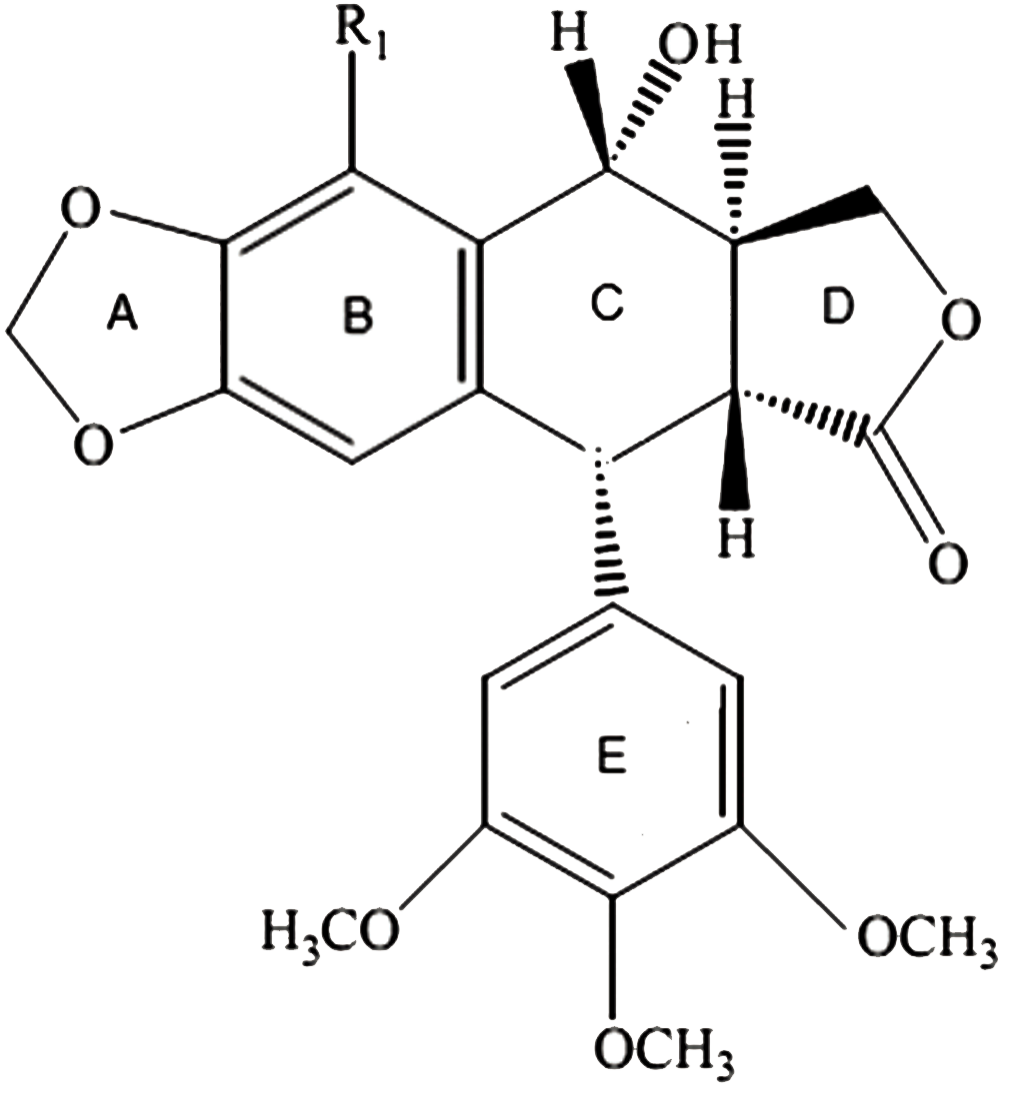
The structures of podophyllotoxin (PTOX, R_1_=H) and 6-methoxy PTOX (MPTOX, R=OCH_3_).

6-methoxy PTOX (MPTOX) is a cytotoxic lignan found in some Linumspecies (17, 18). MPTOX has a structure similar to PTOX, except for an additional methoxy group at the C6 ring B (Fig .1) (19). Based on our knowledge, there is no report of the *in vitro* and *in vivo* biological activities and effects of MPTOX on cancer cells.

Here, for the first time, we investigated the anti-tumor and cytotoxic activity of MPTOX against two human cancer cell lines. The effect of MPTOX treatment on expression of two key genes, TopIIA and Tubulin (*TUBB3*), as key proteins involved in cell division was also examined.

## Materials and Methods

### Materials

To do this experimental study, MPTOX was purified from *Linum album* hairy root cultures as previously described (18). Briefly, extraction of lignans from hairy roots (2 g FW), line R2, were carried out by sonication in methanol (80%) during 1 hour. Dichloromethane and water (1:1 v/v) were added and mixed. The dichloromethane fractions were collected, dried and dissolved in 500 μl of high-pressure liquid chromatography (HPLC) grade methanol and injected into an HPLC (Philips, UV/Vis detector, Pu 41110). The elution solvent was composed of water and acetonitrile with a gradient system (18). A spectrophotometric ultraviolet (UV=290 nm) was used for detection of MPTOX. We performed the analytical separation on a C18-S5ODS3 (250 9 4.6 mm) reversephase (RP) column with a flow rate of 1.0 ml/ minute. The fraction that contained MPTOX was collected, lyophilized and dissolved in dimethyl sulfoxide (DMSO) (Fig .1).

RPMI-1640, fetal bovine serum (FBS), penicillin- streptomycin, and trypsin enzyme were purchased from Gibco (Grand Island, NY, USA). DMSO, Annexin-V-Fluos kit, 3-(4, 5-dimethylthiazol- 2-yl)-2 and 5-diphenyltetrazolium bromide (MTT) were obtained from Roche (Germany). RNXTM-plus solution was purchased from Cinnagen (Iran). The SYBR Green I master mix kit was obtained from Takara (Shiga, Japan) and the cDNA synthesis kit was purchased from Fermentas (Canada).

### Cell culture

The human bladder carcinoma cell line 5637 and myelogenous leukemia cell line K562 were obtained from Pasteur Institute (Iran). Cancer cell lines were maintained in RPMI 1640 (Gibco) supplemented with 10% (V/V) FBS (Gibco), 100 U/ml penicillin and 100 μg/ml streptomycin at 37˚C in a humidified atmosphere with 5% CO_2_.

### Cytotoxicity assay

In order to determine the inhibitory concentration (IC^50^) of MPTOX, we plated the cells at a density of approximately 2×10^4^ cells per well in a 96-well plate. Cells were treated with different concentrations (0-80 μg/ml) of MPTOX, then incubated for 24 hours at 37˚C and 5% CO_2_.

Cell viability was evaluated with the MTT test. Briefly, 10 μl (5 mg/ml) of the MTT dye solution was added to each well for a 4-hour period at 37˚C. After removal of the culture media that contained the soluble MTT dye, fomazan crystals were extracted with DMSO. The absorbance at 490 nm was quantified using an ELISA reader.

We examined the cytotoxic effects of MPTOX in both cell lines with the 10 μg/ml concentration at 24, 48 and 72 hours after treatment. Cytotoxicity was calculated as the concentration of drug that inhibited cell growth by 50% (IC^50^). All experiments were conducted at least in triplicate.

### RNA isolation and cDNA synthesis

Total RNA was extracted from the cells using RNXTM plus solution (Cinnagen, Iran) according to the manufacturer’s instructions. For removal of any genomic DNA contamination, we treated all total RNA with DNase I (Sigma, USA) at 37˚C for 30 minutes. The integrity and concentration of extracted RNAs were examined on agarose gel electrophoresis and with a spectrophotometer, respectively. Reverse transcription reaction for first strand cDNA synthesis was performed with 3-5 μg of purified total RNA with the RevertAid™ Reverse Transcriptase (Fermentas, Canada) using oligo (dT)_18_ in a total of 20 μl reaction mixture according to the manufacturer ’s instructions.

### Real-time gene expression analysis

mRNA expression levels of the *TUBB3* and *TOPIIA* genes were estimated with the appropriate primers. The relative expression of each gene was assessed compared to the housekeeping gene glyceraldehyde-3-phosphate dehydrogenase (GAPDH) with specific primers. The primers for amplification of *TUBB3*, *TOPIIA* and GAPDH were designed using Primer Express software (Applied Biosystems, USA) (Table 1). Quantitative real-time polymerase chain reaction (qPCR) as performed using the 7500 ABI system (Applied Biosystems, Foster, CA, USA) in final reaction volumes of 20 μl with 20 ng cDNA, 10 μl of SYBR Green I master mix (Takara, Shiga, Japan) and 200 nM of the forward and reverse primers according to the manufacturer’s in structions. The PCR reaction was performed as follows:initial denaturation of templates at 95˚C for 3 minutes, followed by 40 cycles of denaturation at 95˚C for 15 seconds and annealing/extension at 60˚C for 30 seconds. Specificity of PCR products was examined on a 2% agarose gel to verify their size and dissociation curve analysis. Different concentrations of cDNA were made for obtaining the efficiency of each primer set. For all gene expression analyses, appropriate negative controls that contained no template were subjected to the same procedure in order to exclude or detect any possible contamination.

The relative gene expression for each gene was estimated by the comparative threshold cycle as described by Livak. Briefly, the mean threshold cycle (mCT) was obtained from triplicate amplification during the exponential phase of the amplification. Then, the mCT value of reference gene of (GAPDH) was subtracted from the mCT values of the *TUBB3*, and *TOPIIA* genes to obtain ΔCT for each gene. After calculation of ΔΔCT values of each sample, the relative expression of each gene was estimated by the ratio formula (ratio=2^-ΔΔCt^) (21). All experiments were conducted at least in triplicate.

**Table 1:** Primer sequences used in this study for gene expression analysis by real-time polymerase chain reaction


Gene names (Accession no.)	Primer sequences	Amplicon size (bp)

*TUBB3 (NM_001197181)*	F: 5΄-ACTACAACGAGGCCTCTTCTCAC -3΄	151
	R : 5΄-TTGTTGCCGGCCCCACTCTGACC -3΄	
*TOPIIA (NM_001067)*	F : 5΄-ATCCTGCCAAAACCAAGAATCG -3΄	174
	R : 5΄-GTACAGATTTTGCCCGAGGAGC -3΄	
*GAPDH (NM_001256799)*	F : 5΄-GTGAACCATGAGAAGTATGACAA -3΄	123
	R : 5΄-CATGAGTCCTTCCACGATAC -3΄	


### Apoptosis assay

We used an Annexin V-PI detection kit (Roche, Germany) to measure the number of apoptotic cells. Both the 5637 and K562 cells at 2×10^5^ densities were suspended in RPMI1640 with 10% FBS, then seeded in two 24-well flat-bottomed plate and incubated for 24 hours at 37˚C . MPTOX and culture medium only were added at 10 μg/ml and incubated for 24 hours. After collection and washing the cells with PBS, propidium iodide (PI) and Annexin V were added directly to the cell suspensions in the binding buffer (10 mM HEPES, 140 mM NaCl, 2.5 mM CaCl_2_, pH=7.4). The cells were incubated in the dark for 15 minutes at 37˚C after which samples were analyzed by flow cytometry. Plasma membrane recognition by positive staining for Annexin V-FITC showed early apoptosis; late apoptotic cells were determined by positive staining for both Annexin V-FITC as well as PI.

### Statistical analysis

The results of cytotoxicity and gene transcriptions were analyzed with one-way ANOVA followed by the t test using Graphpad Prism 5.0 and SPSS (SPSS, Chicago, IL, USA). A P value ≤0.05 was considered significant. Data are shown as mean ± standard deviation (SD).

## Results

### Cytotoxicity assay

**Fig.2:**
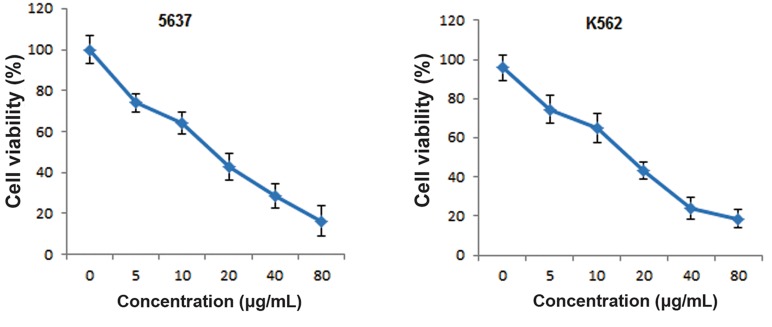
Cytotoxicity effect analysis of 6-methoxy podophyllotoxin (MPTOX) on the 5637 and K562 cancer cell lines. In a dose-dependent manner after 24 hours, MPTOX showed almost the same toxicity, with a lethal dose (LD_50_) of 20 μg/ml on both cell lines. Viability values were determined by MTT assay in triplicate independent experiments (mean ± standard deviation).

To determine the lethal dose (LD_50_) of MPTOX, we exposed both the 5637 and K562 cultured cells in a dose-dependent manner, from 0 to 80 μg/ml, for a 24-hour period. The cytotoxicity assay results indicated that MPTOX significantly decreased cell viability. The LD50 for both cell lines was measured as 20 μg/ml (Fig .2).

**Fig.3:**
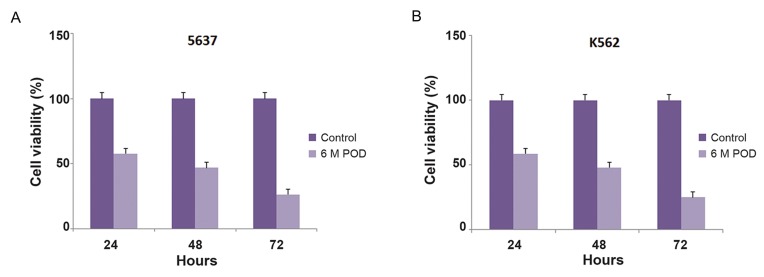
Survival ratios of 5637 (A) and K562 (B) cells treated with 6-methoxy podophyllotoxin (MPTOX). The 80% confluent cell cultures were treated with 10 μg/ml of MPTOX at different times. There was a time-dependent effect of MPTOX in reduction of survival rate in the cells. At 48 hours after treatment, more than 50% of the cells died. Results represent the means of three independent experiments by the MTT assay.

We investigated the biological activity and effect of treatment time with MPTOX by conducting a time-dependent cytotoxicity assay at the non-toxic 10 μg/ml concentration. Cell viability reduced to 60% at 24 hours after treatment. However, the rate of viability reduction reduced following MPTOX treatment of the cells for 48 and 72 hours in both cell lines (Fig .3A, B).

### Real-time gene expression analysis

**Fig.4:**
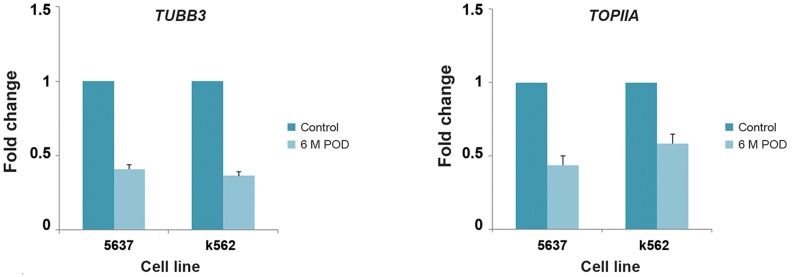
Expression levels of tubulin (*TUBB3*) (left) and topoisomerase II (*TOPIIA*) (right) genes down-regulated in 5637 and K562 cells after treatment with 6-methoxy podophyllotoxin (MPTOX). Each real-time polymerase chain reaction (PCR) examination was carried out at least in triplicate. Data: fold change in relative expression compared with GAPDH on the basis of the comparative threshold cycle [Ct (2^-ΔΔCt^)] method. Values are shown as mean ± standard deviation.

The effects of treatments on gene expressions were analyzed by qPCR. The expression level of GAPDH did not shown any effect by MPTOX treatment at a nontoxic concentration compared to the other examined genes, therefore it was used as an appropriate housekeeping gene for transcription analysis. Expression of the *TUBB3* gene in both cell lines was measured after treatment with 10 μg/ml of MPTOX for 24 hours. The expression levels of both the *TUBB3* and *TOPIIA* genes significantly downregulated in both cell lines compared to control cells. However, the reduction was more obvious for the *TOPIIA* gene expression in K562 cells compared to 5637 cells (Fig .4).

### Apoptosis assay

**Fig.5:**
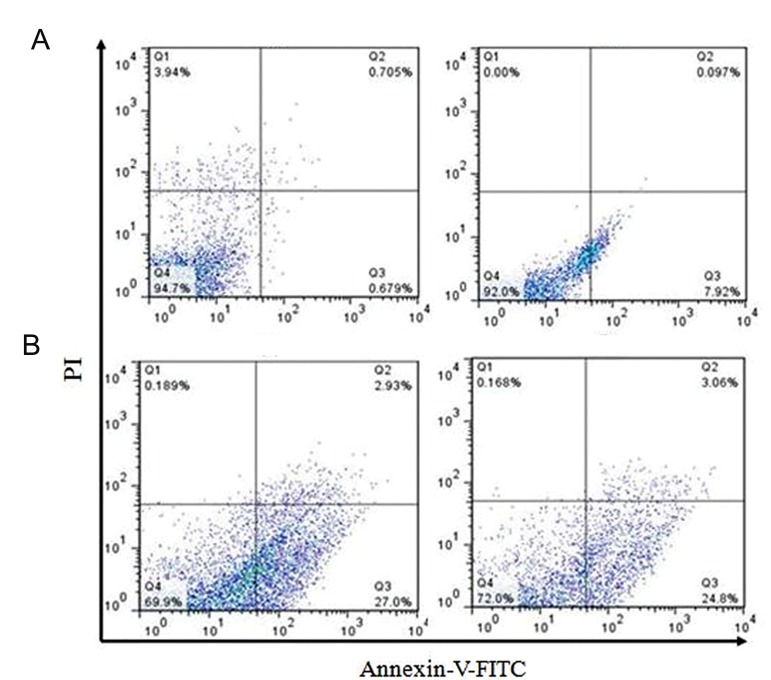
Ability of 6-methoxy podophyllotoxin (MPTOX) to induce programmed cell death in cancerous cell lines. Flow cytometry analysis of 5637 (left) and K562 (right) cells stained with Annexin V-FITC and propidium iodide (PI). A. Untreated cells used as controls and B. Cells underwent apoptosis induced by MPTOX treatment. Treated cells had significantly increased rates of early and late apoptosis compared with the control. For the 5637 cell line, cell cycle analysis showed that the ratio of cells in the G1 phase changed from 39.3 to 20.6%. In the S phase, cells changed from 11.8 to 9.3% and the G2/M ratio decreased from 21.2 to 16.8% with MPTOX treatment. In the K562 cell line, cell cycle analysis showed that the ratio of cells in the G1 phase changed from 31.4 to 22%, for the S phase cells decreased from 13.7 to 10.3%, and for the G2/M ratio the cells decreased from 20.1 to 18.5% with MPTOX treatment. Diagram Q1 to Q4 represents necrotic, early apoptotic, late apoptotic and live cells, respectively.

**Fig.6:**
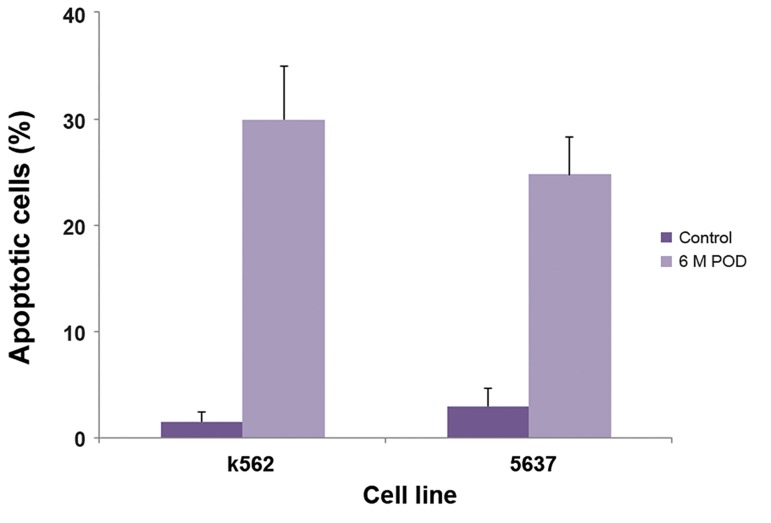
Apoptosis of 5637 and K562 cells induced by 6-methoxy podophyllotoxin (MPTOX) after 24 hours. As shown, apoptosis increased after treatment of cells with MPTOX compared to control cells with no treatment. Values are shown as mean ± standard deviation.

The ability of MPTOX to induce programmed cell death in both cell lines was analyzed by flow cytometry. We treated the 5637 and K562 cells with 10 μg/ ml MPTOX for 24 hours. Untreated cells were considered as control cells. Cells were treated with Annexin V-FITC and PI, then analyzed by flowcytometry. Treatment of the 5637 cells significantly increased early (2.93%) and late apoptosis (27%), while control cells showed 0.679% early apoptosis and 0.705% late apoptosis (Fig .5). In K562 control cells, early apoptosis was 0.97% whereas late apoptosis was 7.92%. However after treatment with MPTOX, early apoptotic cells increased to 3.06% and late apoptotic cells increased to 24.8%. For 5637 cells, the G2/M ratio decreased from 21.2 to 16.8% and for K562 cells the G2/M ratio reduced from 20.1 to 18.5% after MPTOX treatment. Overall, we concluded that total apoptosis remarkably increased compared with control cells following treatment with MPTOX (Fig .6).

## Discussion

The search for new herbal anti-metabolites that have less adverse effects and the ability to induce programmed cell death or apoptosis as treatment of various cancer types is an active area of basic and clinical science research.

Lignans are a family of natural compounds present in many higher plants which are isolated from different species. PTOX, as a popular member of the lignan compounds with different biological activities, is mostly used for production of semisynthetic and more applicable derivatives such as etoposide and teniposide that have less cytotoxicity. It has been shown that PTOX derivatives show great potential in the battle to overcome multidrug resistant cancers by inducing cytotoxicity through multiple mechanisms such as interaction with DNA, along with their decreased adverse effects (20, 21).

In this work, we investigated the biological properties of MPTOX, which has been recently extracted from *Linum album* hairy roots as a PTOX derivative in our research group (18). Cytotoxicity assay showed that MPTOX significantly decreased both K562 and 5637cancer cell viability.Transcription analysis showed that MPTOX reduced the expression levels of the *TUBB3* and *TOPIIA* genes.

A number of agents that contain colchicinebinding sites decrease the expression of class III β-tubulin (*TUBB3*) (22). *TUBB3* is a core member of the beta tubulin protein family that consists of microtubules with alpha tubulin as a heterodimer. This family is in cellular processes such as mitosis, intracellular transport and cell motility (23). Class II β-tubulin (TUBB2) and *TUBB3* are the most frequent isotypes particularly located in epithelial cells which over-express in some cancer cells. Some studies have reported that *TUBB3* plays a role in various cancer types resistant to chemotherapy (24-27).

*TOPIIA* is a key nuclear enzyme involved in the normal replication process. It is one of the main targets for effective anticancer agents such as anthracyclines which bind and block *TOPIIA* activity, followed by inhibition of DNA replication (28).

It has been shown that elevated *TUBB3* and/or *TOPIIA* expression levels in gastric, non-small cell lung cancer (NSCLC), ovarian, cervical, salivary gland and breast cancersis associated with a poorer response to anticancer agents that bind to anti-tubulin agents and poorer prognosis or reduced survival in patients. However, the effect of MPTOX on *TUBB3* and *TOPIIA* gene expressions can be considered apotential agent that may overcome drug-resistance of cancers cells (29-32).

On the other hand, it has been shown that downregulation of *β-tubulin* by Trichosanthin induced apoptosis in HeLa cells (22). Here, our results showed reduced *TUBB3* and *TOPIIA* gene expression levels followed by an increased apoptosis rate. This has suggested that MPTOX also can directly act on transcription levels or indirectly as an inhibitor. However its specific mechanism is not yet known. According to previous studies PTOX, like colchicine, prevents the assembly of microtubules that eventually lead to apoptosis (33, 34). Etoposide- like compounds, in contrast, act on the topoisomerase II enzyme converting it to an irreversible toxin, creating DNA damage which in turn causes cell death (15, 16, 35). Solary et al. (36) have suggested that modification on the B ring increased the anti-cancer activity of PTOX by inhibition of TOPII. Interestingly, we found that MPTOX inhibited the *TOPIIA* as it decreased *TUBB3* expression (19). These resultshave suggested that MPTOX can significantly induce cells to undergo apoptosis, as with other PTOX derivatives (34, 37), probably through both PTOX-like and etoposide-like compounds mechanisms. It is possible that MPTOX may be used as a complementary medicine alongside other routine medicines in order to overcome drug resistance in cancers that express high levels of *TUBB3* and *TOPIIA*.

## Conclusion

Treatment of both 5637 and K562 cells with MPTOX significantly reduced viability and induced programmed cell death. In addition, the expressions of *TUBB3* and *TOPIIA* were suppressed after treatment with MPTOX. Here, we have suggested that MPTOX may inhibit both *TUBB3* and *TOPIIA* gene transcriptions, which possibly cause cell growth arrest and apoptosis. This two-way potential function is suggested to be related to the MPTOX structure in that it has an additional methoxy group. This probably increases cytotoxicity. MPTOX has potential as a novel chemotherapy agent in pharmaceutical studies.
